# Velopharyngeal Stenosis After Adenotonsillectomy in a Child: A Case Report

**DOI:** 10.1155/cris/7184710

**Published:** 2026-07-17

**Authors:** Elisamia Ngowi, Adil Datoo, Mayila Mbuki, Irene Godlove Msamba, Jacqueline Gabone, Christopher Mwansasu

**Affiliations:** ^1^ Department of Paediatrics and Child Health, Aga Khan Hospital Dar es Salaam, Dar Es Salaam, Tanzania; ^2^ Department of Paediatrics and Child Health, Aga Khan University Tanzania, Dar Es Salaam, Tanzania; ^3^ Department of Paediatrics, Kampala International University, Dar Es Salaam, Tanzania, kiut.ac.tz; ^4^ Department of Radiology, Aga Khan Hospital Dar es Salaam, Dar Es Salaam, Tanzania; ^5^ Department of Clinical Pharmacology, Muhimbili University of Health and Allied Sciences, Dar Es Salaam, Tanzania, muchs.ac.tz; ^6^ Department of Otolaryngology, Aga Khan Hospital Dar es Salaam, Dar Es Salaam, Tanzania

**Keywords:** adenotonsillectomy, case report, velopharyngeal stenosis

## Abstract

Velopharyngeal stenosis is a rare condition characterized by partial or complete obliteration of the velopharyngeal port and may present with snoring, obstructive sleep symptoms, and speech abnormalities, typically following oropharyngeal surgery. We report the case of a 6‐year‐old boy who developed persistent diurnal and nocturnal snoring accompanied by recurrent coughing 4 months after adenotonsillectomy. Endoscopic evaluation revealed significant stenosis of the velopharyngeal port. The patient initially underwent surgical release and dilation of the stenotic segment, resulting in temporary symptom improvement; however, symptoms recurred 2 months later due to restenosis. A second surgical intervention was performed with placement of a stent across the stenotic segment to maintain patency during healing. Following stent placement, the patient experienced complete resolution of symptoms, and no further recurrence was observed during follow‐up. This case highlights velopharyngeal stenosis as a rare but clinically significant complication of adenotonsillectomy in children and underscores the challenges associated with its management because of the risk of recurrence. It also demonstrates the potential role of stenting as an adjunctive measure to prevent restenosis and achieve sustained symptom resolution. Awareness of this complication and meticulous surgical technique during oropharyngeal procedures may help reduce its occurrence and improve patient outcomes.

## 1. Introduction

The velopharyngeal port is the passage connecting the oropharynx and nasopharynx. It functions as a muscular valve extending from the posterior surface of the hard palate to the posterior pharyngeal wall. Its borders include the velum (soft palate), lateral pharyngeal walls, and posterior pharyngeal walls. This structure plays a crucial role in creating a secure seal between the velum and pharyngeal walls, separating the nasal and oral cavities. This seal supports essential functions such as speech production, while its opening allows breathing, and its closure prevents food and fluid regurgitation during swallowing [1]. The adenotonsillar tissues are closely associated with the velopharynx. The adenoids are situated on the superoposterior wall of the nasopharynx, while the tonsils are located below the soft palate, nestled between the pillars of the fauces in the oropharynx [2].

Velopharyngeal stenosis refers to the partial obstruction of the space behind the posterior pillars of the velopharyngeal port. It commonly presents with symptoms such as snoring and, in severe cases, obstructive sleep apnea. Due to the velopharynx’s role in speech, stenosis also impairs speech resonance. The condition has both congenital and acquired causes. Congenital causes are rare and result from unknown mechanisms, while acquired causes include trauma and infections. Historically, syphilis was a leading cause of acquired velopharyngeal stenosis and was also implicated in rare cases of congenital stenosis, though it is much less common today [3].

Surgical trauma has been reported as a cause of velopharyngeal stenosis following invasive procedures such as tonsillectomy, adenoidectomy, uvulopalatoplasty, or radiotherapy for nasopharyngeal carcinoma.

Nasopharyngeal stenosis following tonsillectomy results from fibrotic adhesions that narrow the airway and can present with obstructive sleep symptoms, dysphagia, and speech changes [1].

Treatment for this condition is challenging as it is associated with a high incidence of recurrence [2, 4–6].

We present a case of velopharyngeal stenosis, a rare complication of adenotonsillectomy, in a child who had to undergo two surgical interventions to achieve complete recovery.

This case report has been reported in line with the SCARE criteria [7].

## 2. Case Presentation

We report the case of a 6‐year‐old boy with a 3‐month history of persistent snoring, particularly pronounced during sleep or when lying flat. His symptoms had progressively worsened and were accompanied by breathing difficulties, including mouth breathing and frequent coughing. The patient had undergone an adenotonsillectomy 4 months before the onset of symptoms. There was no history of chronic illnesses, and his antenatal history was unremarkable. Immunizations were up to date as per the national immunization program. His developmental milestones were normal for his age.

On physical examination, the patient had a healed tonsillar fossa with postnasal drip. Systemic examination was unremarkable, and his weight and height were within the normal percentiles for his age. Laboratory investigations revealed normal hemoglobin levels and white blood cell counts. A computed tomography (CT) scan of the paranasal sinuses showed regular thickening of the nasopharyngeal soft tissues with no evidence of choanal atresia, as seen in Figure 1. Polysomnography was not performed due to the limited availability of pediatric sleep study services at our institution. Clinical symptoms, imaging, and endoscopic findings were therefore used to assess the severity of the obstruction and guide management.

**Figure 1:**
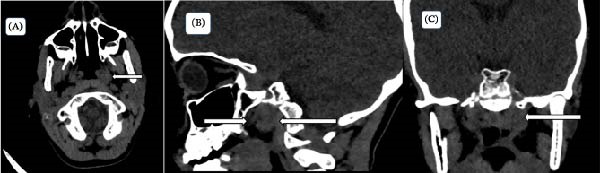
Nonenhanced CT scan of the paranasal of sinuses. (A) Axial, (B) sagittal, and (C) coronal images demonstrate regular thickening of nasopharyngeal soft tissues more in the upper level (white arrows).

Given the history of adenotonsillectomy, nasopharyngeal stenosis or recurrent adenoid hypertrophy was suspected. It was decided to proceed with an examination under anesthesia, diagnostic nasal endoscopy, and a possible adenoidectomy.

Intraoperatively, nasal endoscopy showed a patent choana and nasopharynx with grade 1 adenoid tissue. Endoscopic nasopharyngoscopy through the oropharynx was performed using a 70° 4 mm × 175 mm rigid telescope, showing fibrotic ring stenosis of the velopharynx (Supporting Information 1: Video 1, Supporting Information 2: Video 2, Supporting Information 3: Video 3).

Intraoperative findings revealed an intact choana and grade 1 adenoid hypertrophy. Stenosis was noted at the velopharynx, involving the posterior pillar and the posterolateral surface of the soft palate, measuring ~2.5 cm at its widest diameter, as seen in Figure 2. A thick postnasal mucoid discharge was also observed. The fibrotic stenosis was released endoscopically using monopolar diathermy to restore the patency of the velopharyngeal airway. The naso‐oropharyngeal passage was widened under direct visualization, and hemostasis was achieved. The procedure aimed to relieve the obstruction while preserving the surrounding structures. The patient was stabilized and discharged after 2 days of postoperative care.

**Figure 2:**
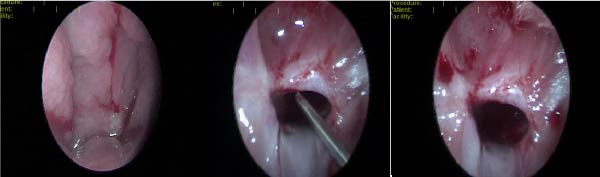
Endoscopic images showing the stenosed velopharyngeal port, and dilation done to release the stenosis.

Upon follow‐up at the clinic, symptoms improved at the 2‐week follow‐up. Unfortunately, 2 months after surgery, the patient returned to the clinic due to recurrence of symptoms, and the patient underwent repeat endoscopic intervention with placement of a stent across the velopharyngeal port to maintain airway patency and prevent readhesion of the stenotic segment.

A customized choanal stent was fashioned from a 4 mm outer diameter endotracheal tube and placed following the procedure to maintain choanal patency. The stent was secured anteriorly with a Prolene suture. The stenting procedure lasted ~30 min, and the stent remained in place for 6 weeks. Postoperatively, the patient underwent regular nasal douching using sodium bicarbonate and sodium chloride sachets to reduce crusting and maintain stent patency. At 6 weeks, the stent was removed in the outpatient clinic under topical local anesthesia by releasing the anterior Prolene suture and gently extracting the stent through the nostril. The procedure was completed without complications, and the symptoms improved significantly thereafter.

## 3. Discussion

Adenotonsillectomy is one of the most common procedures in otolaryngology and is the preferred treatment for obstructive sleep apnea in children [2]. The most common complications of adenotonsillectomy include pain, otalgia, excessive bleeding, and anesthesia‐related issues [8]. Although most complications are immediate, long‐term complications have also been reported, though they remain relatively uncommon [9].

The patient described in this case did not have any of the abovementioned complications after surgery. The complications can be classified into intraoperative, immediate postoperative (within 24 h), delayed (within 2 weeks), and long‐term (occurring weeks to months after the procedure). Delayed complications may include velopharyngeal insufficiency, particularly in patients with a cleft palate or undiagnosed palatal abnormalities. Nasopharyngeal stenosis is a rare long‐term complication typically caused by the approximation of raw mucosal surfaces during the healing process [5, 8, 10].

Our patient presented with symptoms 4 months after the adenotonsillectomy, classifying it as a long‐term complication of the procedure. Large registry data suggest that persistent long‐term complications following tonsil surgery are relatively rare but clinically significant [9].

Most children with velopharyngeal stenosis present with symptoms such as snoring, mouth breathing, and obstructive sleep apnea. Additional symptoms may include dysphagia, chronic otitis media with effusion, rhinosinusitis, and a hyponasal voice [5, 11, 12]. Our patient presented with snoring, mouth breathing, and recurrent cough, which are typical for patients with velopharyngeal stenosis.

In addition to adenotonsillectomy, velopharyngeal stenosis has been reported following procedures such as laser‐assisted uvulopalatoplasty, radiotherapy for nasopharyngeal tumors, and Sutra Neti, an ancient Indian yoga practice [6, 13].

The management of velopharyngeal stenosis is challenging due to its high recurrence rate, with many patients requiring multiple surgeries for complete recovery [4, 5, 14, 15]. It most often occurs as an iatrogenic complication of pharyngeal surgery, and no standardized management approach has been established [16]. Various techniques have been described, including simple dilation of the stenotic velopharyngeal port and the use of local or distant flaps to cover the area after scar tissue removal. Bivalved palatal flaps, which involve a superiorly based flap from the soft palate and an inferiorly based flap from the oropharynx, have been reported as reliable techniques in some cases [2]. There have been reports of complete relief of airway obstruction following the use of facial artery musculomucosal flaps in the treatment of severe velopharyngeal stenosis [17]. A palatal eversion technique has been described in a study involving 12 patients, which was performed in two stages and resulted in a good outcome 1 year postsurgery [2]. Our patient initially underwent simple dilation of the stenotic velopharynx. Unfortunately, due to the recurrence of symptoms approximately 1 month postsurgery, a stent had to be placed to manage the condition.

Other management techniques for velopharyngeal stenosis include steroid injections, balloon dilation of the stenotic segment, carbon dioxide laser release of scar bands, and obturator stent placement [2, 4].

Following the recurrence of symptoms, our patient underwent successful stent placement, after which the symptoms resolved. Recurrence after the initial dilation was most likely due to refibrosis and scar contraction during the healing process, a well‐recognized challenge in the management of velopharyngeal stenosis. Simple release without measures to maintain airway patency may allow opposing raw mucosal surfaces to readhere, resulting in restenosis. The subsequent placement of a stent likely prevented readhesion by maintaining separation of the mucosal surfaces during healing, thereby allowing epithelialization of the widened airway. This highlights the importance of adjunctive measures such as stenting in selected cases to reduce recurrence, particularly in pediatric patients who may exhibit a more robust fibrotic response. Restenosis is often attributed to healing of exposed mucosal surfaces by secondary intention, resulting in scar contraction and readhesion [4, 5, 14, 16].

With the advancement of technology, there have been reports of successful robotic‐assisted bivalved palatal transposition flaps and pharyngeal flap techniques for the treatment of velopharyngeal stenosis [16]. There is no consensus on the best treatment approach for velopharyngeal stenosis due to the limited number of cases and studies as the condition is extremely rare [15]. Therefore, prevention remains a crucial focus in managing this condition.

Objective sleep assessment using polysomnography would have provided a more precise evaluation of airway obstruction severity and postoperative improvement. However, such testing is not routinely available in many low‐resource settings.

This case highlights velopharyngeal stenosis, an exceptionally rare but potentially serious complication of adenotonsillectomy. It underscores the importance of thorough clinical assessment, imaging, and endoscopic evaluation in guiding diagnosis and management, particularly in resource‐limited settings where advanced sleep studies may not be readily available. Given the absence of established management guidelines and the reported risk of recurrence, surgeons should exercise meticulous surgical technique during adenotonsillectomy to minimize the likelihood of this complication and optimize patient outcomes.

## Author Contributions

Elisamia Ngowi was involved in the conception, study design, acquisition, interpretation of data, and drafting of the manuscript. Irene Godlove Msamba and Mayila Mbuki were involved in the literature review of the manuscript. Jacqueline Gabone and Adil Datoo were involved in the interpretation of the radiological data. Christopher Mwansasu was involved in conception, drafting manuscript, interpretation of clinical data and literature review. Christopher Mwansasu is the main guarantor of this research work.

## Funding

No funds were needed to publish this case.

## Disclosure

After using Grammarly, the authors reviewed and edited the content as needed and take full responsibility for the content of the publication. All authors read and approved the final manuscript.

## Ethics Statement

The publication of case reports is exempt from ethical approval at our institution.

## Consent

Written informed consent was obtained from the patient’s legal guardian for the publication of this case report and the accompanying images. A copy of the written consent is available for review by the corresponding author of this article.

## Conflicts of Interest

The authors declare no conflicts of interest.

## Supporting Information

Additional supporting information can be found online in the Supporting Information section.

## Supporting information


**Supporting Information 1** Video 1: Showing nasal endoscopy in which the choana and nasopharynx were patent with grade 1 adenoid tissue.

**Supporting Information 2** Video 2: Showing nasopharyngoscopy through the oropharynx using a 70° 4 mm × 175 mm telescope, showing fibrotic ring stenosis of the velopharynx.

**Supporting Information 3** Video 3: Showing nasopharyngoscopy through the oropharynx using a 70° 4 mm × 175 mm telescope, showing fibrotic ring stenosis of the velopharynx.

## Data Availability

The data that support the findings of this study are available from the corresponding author upon reasonable request.
